# From signal processing of telecommunication signals to high pulse energy lasers: the Mamyshev regenerator case

**DOI:** 10.1515/nanoph-2024-0712

**Published:** 2025-03-28

**Authors:** Christophe Finot, Martin Rochette

**Affiliations:** 131801 Université Bourgogne Europe, CNRS, Laboratoire Interdisciplinaire Carnot de Bourgogne ICB UMR 6303, F-21000 Dijon, France; Nonlinear Photonics Group, McGill University, 3480 University Street, Montréal, Canada

**Keywords:** Mamyshev regenerator, nonlinear signal processing, ultrafast photonics, ultrashort lasers

## Abstract

We look back at many challenges as well as unexpected successes encountered by the Mamyshev optical regenerator, which combines spectral broadening from self-phase modulation followed by offset bandpass filtering. Initially developed for ultra-fast all-optical processing of optical telecommunications signals, the Mamyshev regenerator has become most useful in the field of high-power fiber lasers. Implemented from optical fibers, the Mamyshev regenerator is compatible with integration on an optical chip, and excellent prospects are open for this polyvalent technology.

## 1 Introduction

At the dawn of the twenty-first century, to fulfill the ever-increasing bitrate needs for long-distance transmissions, telecommunications players focused part of their research on all-optical processing technologies, which appeared to be the key to alleviating the accumulation of various types of noise. It is in this context that Pavel V. Mamyshev, working at Bell Labs – Lucent, presented at the ECOC 1998 conference a signal regeneration scheme that now bears his name: the Mamyshev regenerator (MR), patented by its designer [1], [2]. For a decade, this device was the subject of continuous studies and enhancements before optical modulation formats slowly transited from the simplest intensity on–off keying to coherent modulation formats involving phase and multilevel amplitudes. While the data regeneration work has shifted from pure amplitude regeneration to a combination of amplitude and phase-regenerating technologies, pulse regeneration has been the subject of a dazzling renewed interest in the last decade, but in a very different application field, namely the generation of high power and ultrashort pulses. In this field, the MR has gradually established itself as a key element in breaking records fiber architectures.

This contribution does not aim to provide an exhaustive review of the numerous works that exploit MR, but rather to demonstrate how knowledge gained from one field can be leveraged to achieve success in another. Thus, we will discuss the principle of the MR device, its evolution in the telecommunication and fiber laser worlds, and its prospects.

## 2 Principle of the Mamyshev regenerator

**Figure 1:**
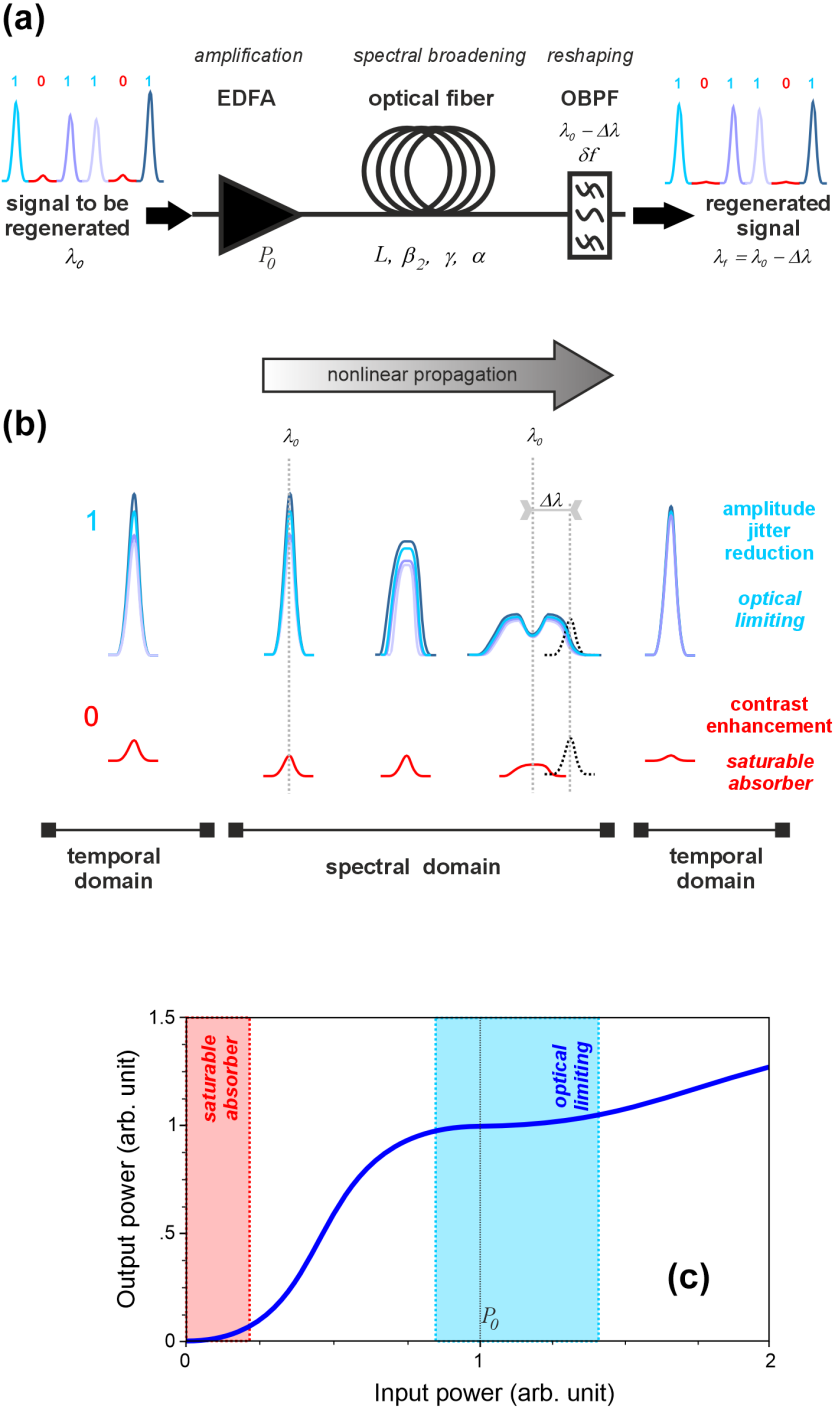
Principle of the Mamyshev regenerator. (a) Schematic of the Mamyshev regenerator and associated variables; EDFA – erbium-doped fiber amplifier; OBPF – optical bandpass filter. (b) Input and output pulses with different power levels are shown in the temporal domain, while their evolution within the nonlinear segment is shown in the frequency domain. (c) Typical transmission function of the MR.

The working principle of the MR is relatively simple, which makes it a device that is elegant and attractive to implement. The pulse reamplification and reshaping (2R) principle is summarized in Figure 1a. A binary signal is composed of pulses representing a digital “1,” while no pulse represents a digital “0.” Consider this signal being contaminated by noise after a long-distance propagation containing several cycles of lossy propagation in optical fibers followed by noisy amplification. The resulting noisy signal then contains digital “1” that are pulses of variable amplitude and duration, and digital “0” that takes the shape of low-power fluctuations. In the regeneration process, each optical pulse centered at wavelength *λ*_0_ is amplified and propagated into a nonlinear optical fiber (or any other nonlinear Kerr element) where it experiences an intensity-dependent self-phase modulation and subsequent spectral broadening (see Figure 1b) [3], [4]. After nonlinear processing, an optical filter is cutting through the spectrally broadened spectrum at a wavelength that is spectrally offset by Δ*λ* with respect to *λ*_0_. The shape of the filter determines the properties of the emerging pulse, resulting in pulse reformatting of the digital “1,” with an identical pulse amplitude and duration. The scenario is different for noisy digital “0.” The low-power noise experiences negligible spectral broadening into the nonlinear fiber and is mainly rejected by the offset filter, leading to a clean digital “0.” Such behavior ensures a high-contrast saturable absorber function, with a preferential attenuation of the noise while providing a good transmission of the pulses. The resulting extinction ratio is easily adjusted by tuning the offset filter. A larger filter offset leads to a better extinction ratio but at the cost of a higher power requirement from the amplification stage.

Since Kerr nonlinearity is an instantaneous process with respect to the duration of a telecommunication pulse, the regenerator does not suffer from any limited response time. This is an undeniable strength compared to saturable absorbers based on material absorption whose response-time struggle to keep up with the increase in telecommunications speed, which also often require higher operating powers. Such saturable absorbers also suffer from limited reproducibility and problematic aging.

The supplementary asset of the Mamyshev device is that, in addition to acting as a saturable absorber, it also regenerates the level of logical “1,” acting as an optical limiter to moderate the amplitude jitter of pulses forming the return-to-zero data stream. This results from the presence of a plateau on the transfer function linking the output power to the input power (Figure 1c). Along with the Kerr effect, normal chromatic dispersion is necessary to shape this plateau and to avoid issues such as pulse compression and splitting that occurs in the anomalous dispersion regime [5]. Note that the temporal broadening and peak pulse’s flattening experienced upon nonlinear propagation in a normally dispersive fiber [6] (but without spectral filtering) was an approach tested to open the eye diagram of a data stream and enhance the detection margin at the end of the transmission line [7].

Thus, thanks to its qualities, the MR was part of a family of nonlinear pulse regenerator approaches that were studied alongside the Nonlinear Optical Loop Mirror (NOLM) [8], [9], the spectral filtering of solitons [10], [11], and four-wave mixing-based devices [12], [13]. Nonlinear polarization rotation has also been shown to discriminate high-intensity pulses from residual sidelobes [14], but the powers involved were not in line with usual telecommunication requirements. Finally, we can mention, as part of these Kerr-based techniques, a sister approach of the MR where instead of considering self-phase modulation, nonlinearity is involved through cross-phase modulation acting on a continuous seed. Under the action of the cross-phase modulation, the continuous wave broadens, and bandpass filtering eliminates the continuous part, giving birth to a wavelength-converted replica of the initial signal with an improved extinction ratio [15], [16], [17].

## 3 Demonstration of the regenerative properties for all-optical processing of a data stream

The MR was initially proposed in the context of an experimental demonstration that showed a visible improvement on both the pulse extinction ratio and the amplitude jitter [1]. This first proof of principle was based on a degraded 10 Gbps signal undergoing phase self-modulation in an 8 km long normal dispersion fiber. The following decade saw progress in many directions.

A first line of research consisted in better understanding the various features, optimal operating conditions, and limits of the device. Indeed, the MR offers many degrees of freedom (see Figure 1a): dispersion *β*_2_, nonlinearity *γ*, attenuation *α*, and length *L* of the nonlinear Kerr medium, the operating power *P*_0_, or the spectral shift Δ*λ* of the optical bandpass filter of bandwidth *δf*. In this multivariate context, researchers must rely on modeling tools to optimize the regenerator response. As the physical parameters of the fibers and filter are usually characterized with high accuracy at telecommunication wavelengths, it is possible to rely on numerical modeling of the device based on the nonlinear Schrodinger equation [18] without any unknown parameter. The numerical algorithm being efficient and the predictions being quantitative, systematic simulations of the multivariate problem have been performed, and design rules have thus been proposed. A link between the physically measured performances [19], [20] and the phenomenon of wave-breaking that appears in a normal dispersion fiber [21] has been established. Other works have highlighted the ability of the MR to discriminate the noise from the signal [22]. This unique property enables the improvement of the bit error ratio and makes the MR scheme a superior candidate for all-optical regeneration [23]. Complementary studies have focused on the limitations of MRs, highlighting an increase in timing jitter, or care needed to avoid temporal pulse overlap and subsequent interactions between two adjacent pulses in the spectral broadening phase [24].

Another challenge for the development of the MR was to lower the required pulse power for nominal operation and reduce the required nonlinear fiber length. This was achieved using specialty nonlinear fibers with larger nonlinear coefficients *γ*, such as commercially available fibers [25] or microstructured fibers [26] that appeared in the early 2000s, thanks to their design that included a reduced modal surface area. Glasses made of materials other than silica, such as bismuth [27] and chalcogenide [28], stimulated research into new chemical compounds that increase the nonlinear coefficient by several orders of magnitude. The increase in nonlinear effect is, however, sometimes accompanied by unwanted two photons absorption [29], [30].

**Figure 2:**
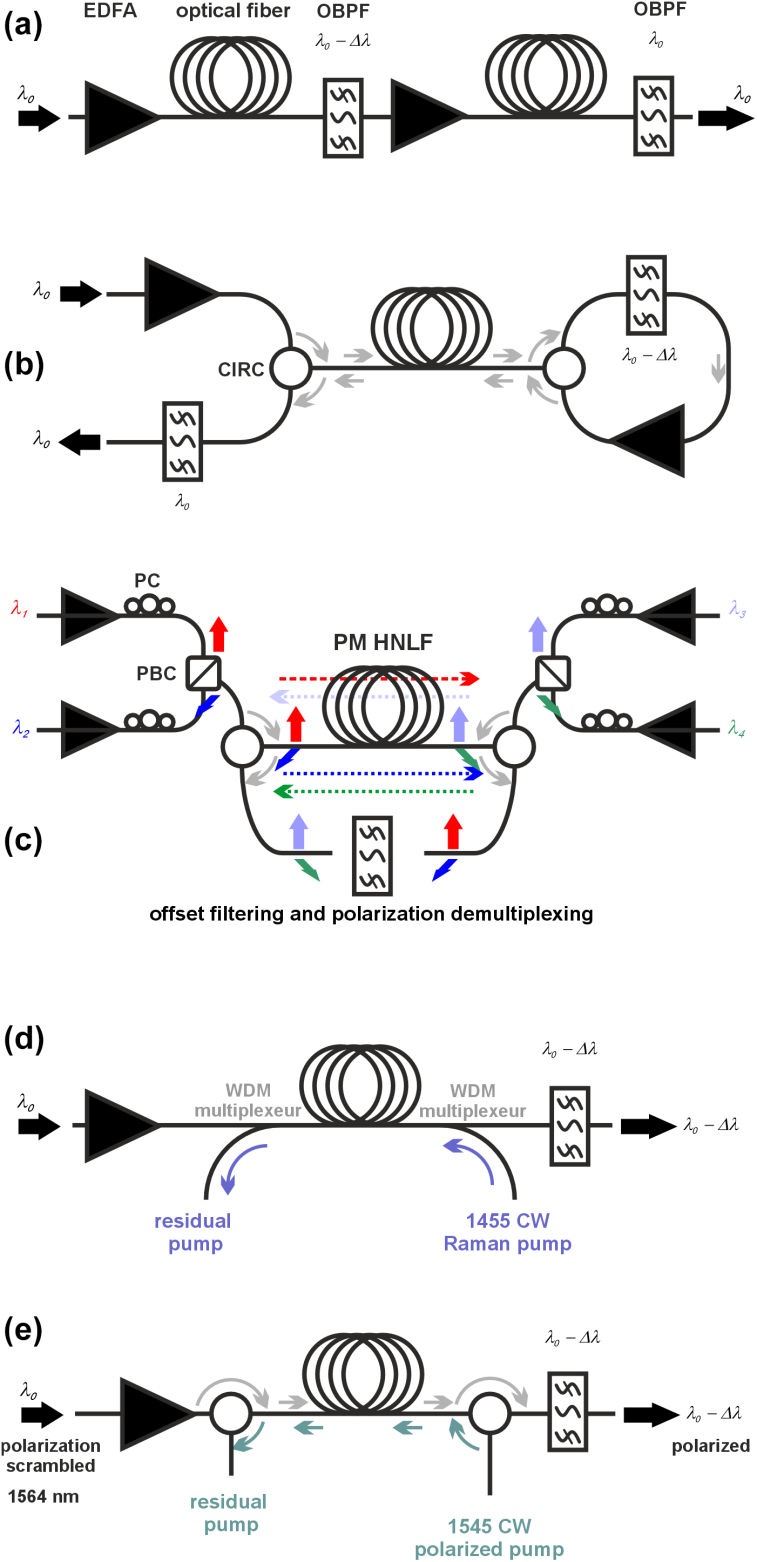
Some evolutions of the MR: (a) double stage device aimed at restoring the initial wavelength. (b) Double-pass configuration, (c) 4 wavelength regenerator, (d) MR including distributed Raman amplification, (e) MR combined with repolarization process. EDFA – erbium-doped fiber amplifier; OBPF – optical bandpass filter; CIRC – optical circulator; PM HNLF: Polarization maintaining highly nonlinear fiber; PBC – polarization beam combiner; CW – continuous wave. Setups have been discussed in [34], [38], [41], [42].

The practical interest of Mamyshev regeneration lies in its potential use at high repetition rates, to overcome the data rate bottleneck induced by traditional regeneration schemes involving conversion between the optical and the electrical domain, electrical regeneration, and conversion from the electrical to the optical domain. The work has thus demonstrated the compatibility of the technique with data rates exceeding 40 Gbit/s [31], [32], [33], [34], [35] and up to 160 Gbit/s [36]. A key issue of the MR was the incompatibility of simultaneously managing several wavelength-division multiplexed (WDM) channels. Indeed, if the MR is based on intrachannel nonlinearity, it must not be affected by cross-nonlinearity from another communication channel. This could be somewhat mitigated by exploiting a counterpropagative configuration [37] and by using polarization maintaining fibers [38] (see Figure 2c). Up to four WDM channels could be processed in parallel. A more comprehensive approach, scalable to any number of WDM channels, involves carefully chosen group-delay management [39], with simultaneous regeneration of up to 16 channels demonstrated [40].

By design, the MR naturally performs wavelength conversion, as well as offering the option to convert the output pulse duration from an appropriate selection of *δf*. If the intended application requires avoiding wavelength conversion, then a two-stage MR architecture with spectral offsets of the same magnitude but opposite signs fulfills this requirement (Figure 2a). This option further strengthens the noise suppression and optical limiting features of the device [35]. To limit the cost and space requirements of several nonlinear fibers, the double-pass configuration is a cost-effective option that has been successfully tested [34] (Figure 2b). Such a bidirectional setup requires cautious management of potential crosstalk induced by Rayleigh or Brillouin backscattering [37]. Just like the NOLM device, which can include an amplification function [43], it is possible to combine nonlinear spectral broadening with a distributed Raman amplification [41] to increase the power level after filtering [44] (see Figure 2d). In the presence of gain, nonlinearity, and normal dispersion, any pulse tends before filtering to the same parabolic self-similar profile that is an attractor of the system [45], [46]. The addition of other features has also been implemented to mitigate impairments induced by polarization mode dispersion [31], [47] or even completely regenerate the polarization state thanks to the polarization attraction dynamics [42] (see Figure 2e). A subsequent resynchronization operation can also be added to MRs [33], [36].

Overall, the work carried out until the mid-2000s enabled a detailed understanding of the MR device and convincing experimental proofs of concept. The MR concept was even tested in an on-field telecommunication system to perform regeneration of a 160 Gb/s return-to-zero signal [36]. Every single challenge that was thrown at MR has been overcome when dealing with on–off keying format. Nevertheless, the introduction and fast deployment of coherent communication systems after 2007 led to a major paradigm shift. Handling or preserving the optical phase, as well as multiple levels, was a radical change where the MR, a solution compatible with amplitude return-to-zero modulation systems, could hardly fit. Thus, after nearly a decade of intensive research attention, the interest in MRs dropped and nearly faded away at the beginning of the 2010s. To address the increased complexity of data format, a path that has been explored is to couple the MR capabilities with other building blocks able to perform phase regeneration [48]. For some time, other all-optical regeneration approaches based on parametric mixing [49], [50] or on NOLMs [51], [52] have been investigated with some promising achievements in manipulating or preserving the phase. However, the advent of digital signal processing (DSP) and, more recently, artificial intelligence compensation schemes have taken the lead industrially and commercially over optical nonlinear-based signal processing devices. If long-haul telecommunications have definitively adopted coherent modulation formats, and the intensive use of DSP units, future opportunities may appear in the context of sprawling hyperscale data centers where on–off keying remains an attractive format. Therefore, stimulated by the demonstration of WDM-compatible solutions, there remains some academic research dedicated to innovative all optical-regeneration solutions for amplitude only or phase and amplitude formats.

## 4 Toward the concept of a regenerative self-pulsating source

**Figure 3:**
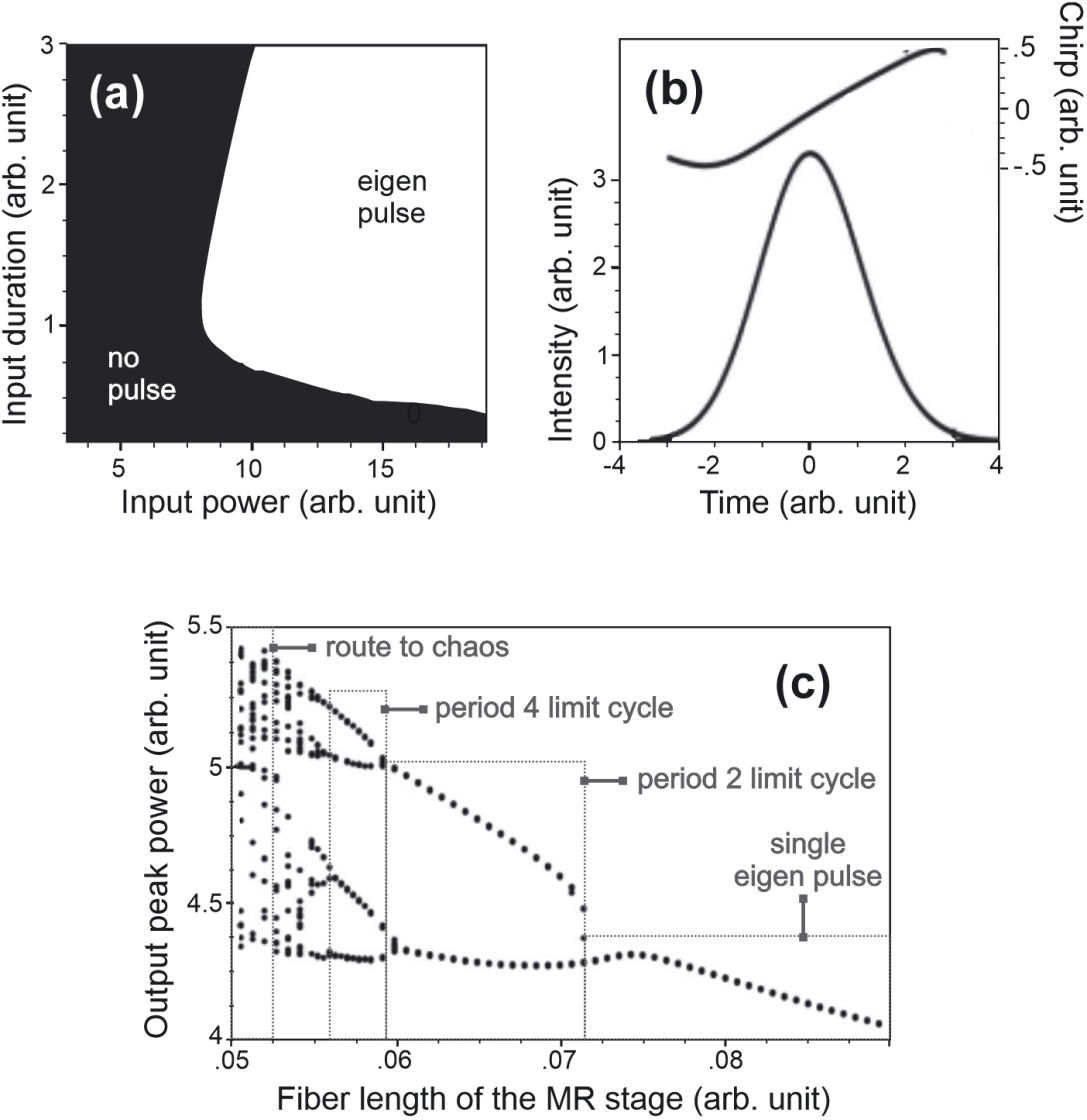
Properties observed after propagation in a chain of MRs. (a) Depending on the input pulse peak power and duration, the pulse disappears (black region) or converges toward the eigen pulse (white region). (b) Example of eigen pulse: the temporal chirp and intensity profile. (c) Peak power of the eigen pulses according to one property of the MR, i.e., the nonlinear fiber length. Results are adapted from [54].

While the MR’s prospects seemed bleak, new studies have opened a very different path. In 2007, Pitois et al., demonstrated numerically that from an initial optical noise background, the concatenation of many double-stage MRs naturally gave rise to a well-defined pulse, called eigen pulses of the system [53]. After a few paired MRs, the transfer function becomes nearly a step-like function. Consequently, after propagation in numerous cascaded MRs, any pulse will either disappear or converge to the properties of the eigen pulse. The many variable peak power and duration of input pulses to the cascaded MRs leads to well-defined basins of attraction as shown in Figure 3a. The eigen pulse is the pulse observed at the output of cascaded MRs and leads again to the same eigen pulse when input to a double-stage MRs (see an example in Figure 3b). Depending on the chosen parameters of the MRs, other more complex behaviors such as basins of attraction with fractal borders, limit cycles and/or routes to chaos through period doubling, appear. Such behaviors can be analyzed from the perspective of nonlinear dynamics with bifurcation diagrams (Figure 3c) [54]. One way to set up an infinite cascade of MRs is to use a double-stage MR configuration in a cavity.

**Figure 4:**
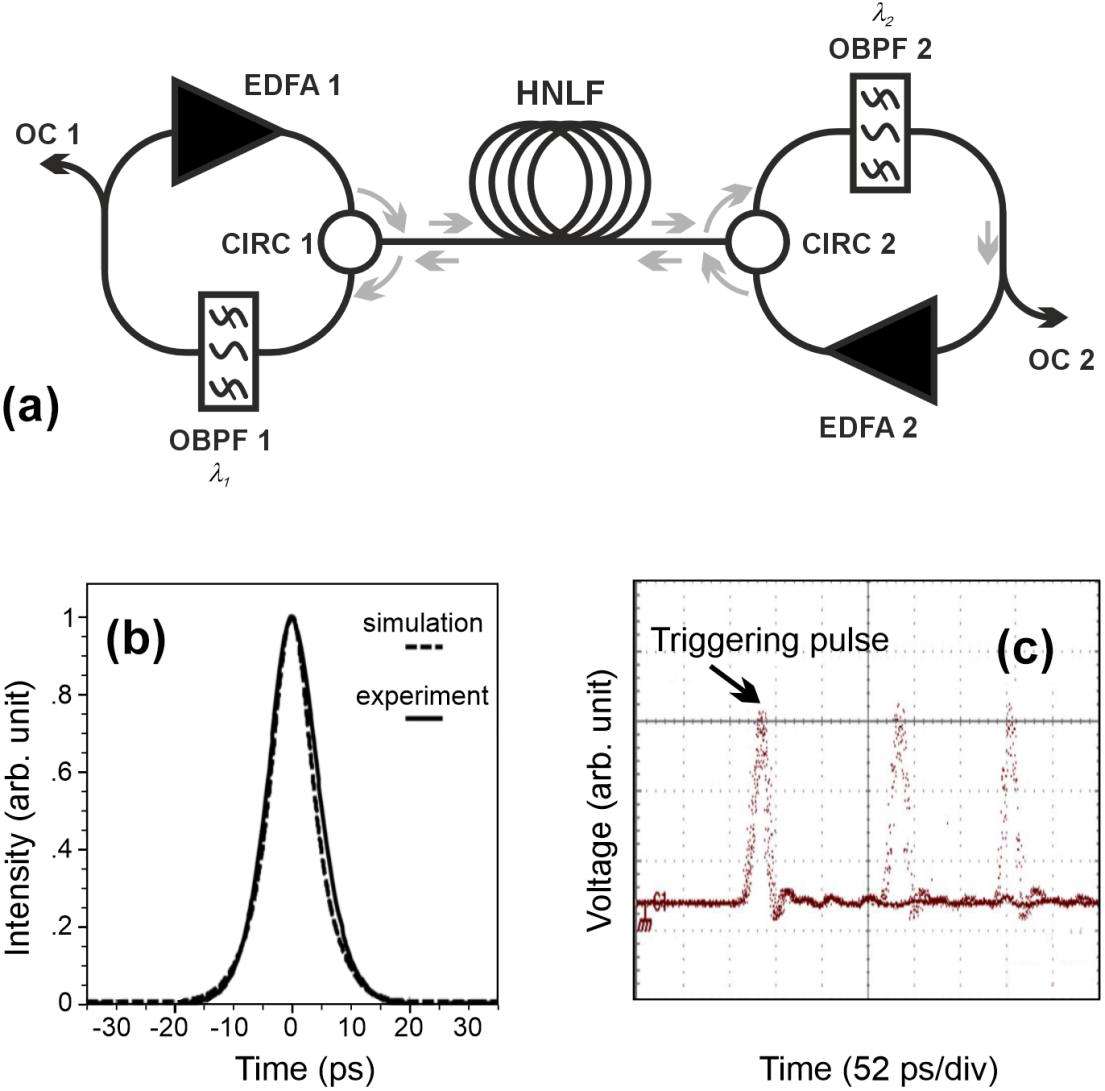
Regenerative self-pulsating source. (a) Experimental setup of the self-pulsating cavity proposed by Rochette et al. EDFA – erbium-doped fiber amplifier; OBPF – optical bandpass filter; CIRC – optical circulator; HNLF: 1-km normally dispersive highly nonlinear fiber; OC – optical output coupler. (b) Intensity autocorrelation profile of the output pulse. (c) Oscilloscope trace stressing that the generation of the pulse appears aperiodically. Results are adapted from [55].

M. Rochette and coworkers experimentally implemented the concept of cascaded MRs and observed the spontaneous formation of eigen pulses [55]. For the sake of compactness, the pulsed source was implemented using a 1-km long highly nonlinear fiber used in a bidirectional configuration (Figure 4a). At the output of the device, pulses with a clearly defined and quasi transform limited shape were emitted (Figure 4b). The cavity could operate in a self-pulsating regime, where several pulses would spontaneously populate the optical cavity. When the bandpass filters were offset sufficiently with respect to each other, the cavity could also be prevented from spontaneously forming pulses. However, in this so-called pulse buffering regime, one or several pulses could be injected in the cavity and remain in circulation indefinitely. In the self-pulsating regime, tens of thousands pulses per cavity can coexist with aperiodic spacings (Figure 4c) [56], [57]. The system, therefore, had the interesting feature of emitting perfectly defined pulses without having the usual signatures of mode-locked lasers such as those operating at the repetition rate imposed by the optical cavity length. Higher-order effects such as Raman self-frequency shift can also come into play to trigger self-pulsation of the cavity [58]. Self-pulsation can also take the shape of a single noisy pulse that circulates in the cavity [59]. The broad pulse is formed as a burst of eigen pulses separated by random pulse to pulse delays [59].

## 5 Mamyshev regenerator as the key to high-power fiber laser sources

### 5.1 Use as a pulse enhancer

Thanks to its ability to generate clean pulses, eliminating the pedestals through its function as a saturable absorber, while allowing the pulses to be reshaped thanks to its spectral filtering, the MR has seen its applications go beyond the framework of data stream processing to spread in the field of ultrashort optical sources.

One of its first uses was again in the field of telecommunications, as one of the building blocks of an optical source with the target to generate, from an initial electrooptic phase modulated continuous wave or an ergo laser running at 10 GHz, a high-quality pulse train suitable for optical time domain multiplexing above repetition rates of 640 Gbit/s [60], [61], [62]. Thinking in terms of MRs processing can also shed new light on the performance of multiwavelength sources obtained after optical bandpass slicing of a continuum generated in the normal dispersion regime [63], [64].

Another relevant application was demonstrated by Fu et al. who used an MR in a high-power amplification chain to significantly improve the quality of a pulse from a gain-switched laser diode [65]. If the initial 10-ps pulses were of poor quality and unattractive for practical application, after MR and multistage amplification, durations below 150 fs and peak powers exceeding 10-MW were achieved. Some other results demonstrate in a single MR the compression of 80 ps pulses down to 3 ps at 1,064 nm [66] or the compression of 60 ps pulses down to 7 ps at 914 nm [67]. MRs were also involved in the management of the amplified spontaneous emission in a chain of ytterbium amplifiers [68].

Without explicitly mentioning the term MR, other experimental demonstrations have highlighted the benefits that could be derived from spectral broadening by self-phase modulation of mJ pulses in an argon-filled hollow-core fiber followed by spectral filtering [69]. Filtering the outmost lobes in the broadening spectrum leads to measured temporal contrast enhancement by seven orders of magnitude. With such highly energetic pulses and as the optical limiting aspect of MRs is not the target, the propagation can be nearly purely nonlinear without minimal dispersion effects. One of the applications has been for bandwidth tunable and frequency shifting of ultrashort pulses [70], [71]. The possibility to generate femtosecond two-color output with a high level of synchronization [72] is particularly attractive, with practical applications in nonlinear spectroscopy and microscopy [73].

### 5.2 The main component of breaking record fiber architecture

The last section of Pitois’ numerical work explored the possibility of transposing MRs within a fiber laser [54]. The idea of combining spectral broadening with frequency-shifted filtering was also numerically present in an SPIE conference proceeding by M. Piché (sadly passed away in December 2024). It was in 1993 [74], five years before the explicit proposal of MR. Mamyshev’s technique then joins other artificial saturable absorbers exploiting Kerr nonlinearity, such as NOLM or its gain-including variant [75], the nonlinear polarization rotation [76], or the soliton stabilization technique where an intracavity bandpass filter favors mode-locked operation [77].

**Figure 5:**
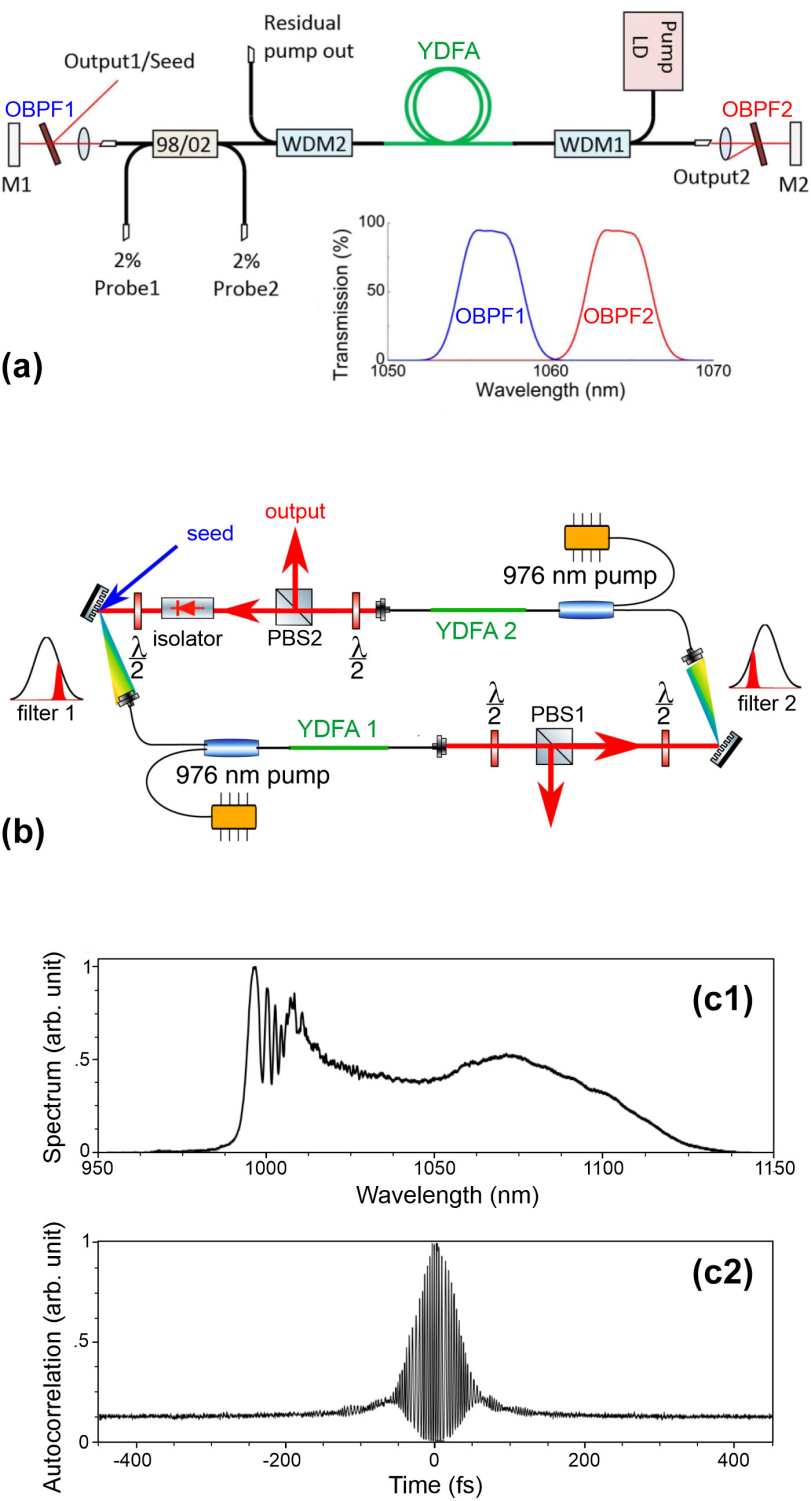
Mamyshev regenerator as the key of a new high-power fiber laser architecture. (a) Schematic diagram of the first experimental Mamyshev laser architecture. YDFA – ytterbium-doped fiber amplifier; OBPF – optical bandpass filter; LD – laser diode; M – mirror; WDM – wavelength division multiplexer. (b) Experimental setup used in the work of Wise’s group; PBS – polarization beam splitter. (c) Output pulse properties for a pulse energy of 49 nJ: optical spectra and autocorrelation. Results are adapted from [78] and [79].

However, the transition of the telecommunications world to the mode-locked laser has not been trivial. Indeed, in the laser community, the accumulation of a large nonlinear phase is viewed as a risky scenario to be avoided. Thus, the use of fiber lengths that are shorter by several orders of magnitude imposed a corresponding increased intracavity peak power, which was made possible by switching to ytterbium wavelengths and the use of femtosecond rather than picosecond pulses. Therefore, it was not until 2015 and the work of Regelskis et al. [78] that an MR-based mode-locked laser with energies above the nJ in a linear cavity configuration appeared in the literature (see the experimental setup in Figure 5a). Only 2 years later, an article by Liu et al. [79] struck a blow by demonstrating the generation of 50-nJ pulses with a duration of only 40 fs after external compression. This results in peak power reaching the MW level (see panels (b) and (c) of Figure 5).

These publications then generated a craze and triggered many new achievements. The concept of high energy Mamyshev laser was extended to spectral bands other than ytterbium, such as erbium [80], thulium [81], [82], and more recently with neodymium [83]. Reliable all-fiber architectures are possible [82], [84], [85]. Extensions to solid-state oscillators [86] and multimode operation [87] have also been encompassed. The architecture can also sustain the generation of cylindrical beams [88]. Solutions have rapidly gained in maturity and the strong rejection of noise provided by the spectral offset filtering provides high environmental stability, especially when normally dispersive amplifiers sustaining self-similar propagation are used. The highly dissipative nature of this architecture where a significant portion of the pulse energy is lost through the virtual saturable absorber has not been found to be a major issue. Efficiencies of the laser of several tens of percent have been experimentally recorded: as two examples, average output powers of 320 mW and 1.5 W have been recorded for pump powers of 1,050 mW [85] and 5.6 W [89], respectively. The parameters of the pair of bandpass filters (bandwidth, frequency offset, and shape) are crucial for an efficient and stable operation [90], [91], [92].

The flip side of the Mamyshev laser is a non-self-starting nature. Different strategies have been implemented to overcome this difficulty: including adding a starting arm [93], modulation of the pump and tuning of the filter [94], [95], using nonlinear polarization rotation [96], or seeding with passively Q-switched microlasers [97], [98]. Controllable intracavity pulse shaping [99] was also proposed. We invite the reader to refer to some recent reviews to have an overview of the rather impressive increase in performance of this type of architecture, which is now becoming one of the potential rivals to Titanium Saphire solutions if a wide wavelength tunability is not sought [100], [101], [102]. Durations down to a few cycles can thus be generated [96], and optical spectra spanning several hundreds of nanometers have been obtained around ytterbium [96] and erbium [103] wavelengths. Pulse energies above the µJ are reached on table-top experiments, leading to peak power above 10 MW directly from the fiber oscillator [104], [105]. Hollow core fibers can also be involved to decrease the repetition rate down to the MHz without compromising the pulse quality [106].

It should also be noted that many dynamics observed in more traditional fiber architectures without wavelength conversion have also been reported in the Mamyshev laser, which is now part of the large family of highly dissipative solitonic structures. About these different characteristics, we can mention the harmonic mode-locking operation [107], [108], [109], the subharmonic operation with the presence of breathing structures and route to chaos [110], [111], [112], formation of bound pairs [82], [113], [114] or interaction of pulses [115], and coherence memory and amnesia [116] to cite only a few.

## 6 Toward a device on the chip scale

With the application potential initially hoped for in the field of optical telecommunications, the question of miniaturization of the device and the implied reduction in power quickly emerged. One of the most remarkable solutions was to turn to optical chips, which made it possible to solve several problems simultaneously, starting with a smaller spatial footprint. The better confinement of light also makes it possible to reduce the effective area of the mode, thus accentuating the consequences of Kerr nonlinearity. An adequate waveguide design optimizes its dispersive properties, allowing it to operate in a favorable nonlinearity to dispersion ratio. Finally, the reduced propagation length intrinsically limits the component latency time compared to a long fiber.

**Figure 6:**
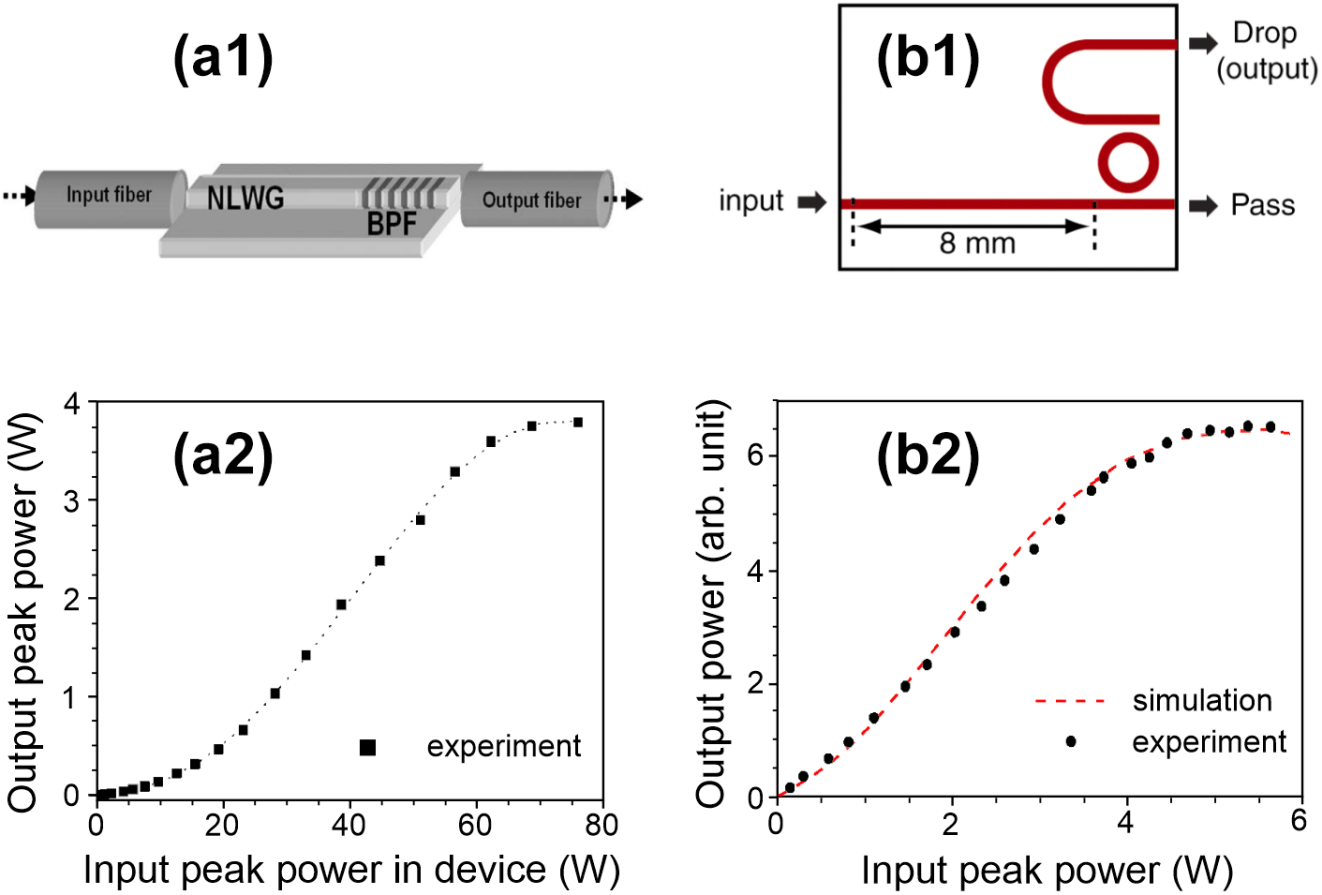
On chip MR devices (a) on a chalcogenide chip, (b) on a silicon chip. The component architectures are sketched in panels (1), and the resulting transfer functions are provided in panels (2). Results are adapted from [119] and [120].

Sydney University in Australia has been a pioneer in this direction, with advanced work to identify the potential of single-mode waveguides made of chalcogenide glasses [117], [118], [119] (see Figure 6a1 for the component). Chalcogenide has a high intrinsic nonlinearity, several thousand times larger than silica, with a normal dispersion favorable to the operation of the Mamyshev device. Thus, the functionalities demonstrated on fibers from several meters to several hundred meters could now be transferred to an optical component with the typical response function of the MR (see Figure 6a2). The spectral filtering functionality was also embedded within the component thanks to integrated Bragg gratings.

Other materials were also tested, including a silicon platform. On the same component, the spectral broadening stage in an 8 mm long waveguide is combined with spectral filtering based on a ring resonator [120] (see Figure 6b1). Again, the expected regeneration function was observed (Figure 6b2) and can be accurately predicted using numerical simulations. However, two-photon absorption and the free-carrier effect can affect the dynamics [121]. Integration studies have stagnated since then, while advances in manufacturing techniques now make it possible to achieve low losses, thereby raising hopes for significantly increased nonlinear propagation lengths and reduced power consumption. Material less impacted by two-photon absorption such as silicon-rich nitride has also emerged at telecommunications wavelengths [122], [123]. Moreover, silicon-nitride-based waveguides can be meter long and handle watt-level average powers for nonlinear processing [124]. Opportunities are, therefore, to be seized in this area, which has been done, for example, in the case of NOLM, demonstrated on a chip with submicrometric transverse dimensions [125]. The advent of on-chip mode-locked lasers [126], [127] also opens several interesting avenues for the realization of an on-chip Mamyshev laser. Even though the expected powers will not compete with those achievable in optical fibers that inherently offer better heat dissipation, these on-chip Mamyshev lasers should soon deliver high-quality femtosecond pulses at high repetition rates.

## 7 Conclusions and perspectives

In this nonexhaustive overview and through the historical development of this research theme, we have attempted to show how a technology initially thought out and developed for about 10 years by and for the actors of the field of optical telecommunications has not succeeded in breaking through on its initial target, in particular, because of the radical change in the landscape of this field. Nevertheless, this concept, which could have been abandoned and forgotten, found its outlet a few years later, namely the field of ultrashort sources. P. V. Mamyshev has authored a conference proceeding and a patent on this topic, but the concept he introduced has been remarkably fruitful and finally became a game changer by presenting the most promising class of ultrafast fiber oscillators.

Other lessons can be drawn from the Mamyshev regenerator history, such as avoiding the compartmentalization of application fields that can, in the end, intersect to give great results. Thus, while one of the significant challenges in telecommunications research was to reduce the operating power well below the Watt, the MR-based laser has proven its usefulness in the generation of MW pulses. While the original purpose of the MR concept was the processing of information at high bit rates, the objectives are now the use of such sources in fields such as nonlinear imaging or materials processing. It is also interesting to note that the relative complexity of the MR, i.e., the use of two filters offset in wavelength, is in no way an obstacle to practical and efficient use.

With the progress of materials and designs at the scale of an optical chip, an application path could open to bring forward the MR ability to process information at a potentially unlimited bandwidth. Indeed, having a nonlinear transfer function is one of the key elements of an artificial neuron. The combination of nonlinearity offers such a possibility that would deserve to be explored for machine learning oriented applications, keeping in mind all the degrees of freedom already demonstrated in the other domains that allow potential in parallel.
